# Protective Effects of Six Selected Dietary Compounds against Leptin-Induced Proliferation of Oestrogen Receptor Positive (MCF-7) Breast Cancer Cells

**DOI:** 10.3390/medicines4030056

**Published:** 2017-07-26

**Authors:** Meran Keshawa Ediriweera, Kamani Hemamala Tennekoon, Sameera Ranganath Samarakoon, Ira Thabrew, E. Dilip de Silva

**Affiliations:** Institute of Biochemistry, Molecular Biology and Biotechnology, University of Colombo, 90, Cumaratunga Munidasa Mawatha, Colombo 00300, Sri Lanka; kamani@ibmbb.cmb.ac.lk (K.H.T.); sam@ibmbb.cmb.ac.lk (S.R.S.); irathab@gmail.com (I.T.); edilip.desilva@gmail.com (E.D.d.S.)

**Keywords:** breast cancer, obesity, leptin, dietary compounds

## Abstract

**Background:** Obesity is considered as one of the risk factors for breast cancer. Leptin has been found to be involved in breast cancer progression. Therefore, novel approaches to antagonize biological effects of leptin are much needed. The objective of this study was to evaluate the protective effects of six dietary compounds (quercetin, curcumin, gallic acid, epigallocatechin gallate (EGCG), ascorbic acid and catechin) and assess the phosphorylation of extracellular signal-regulated kinase 1/2 (ERK1/2) in leptin-stimulated MCF-7 breast cancer cells in vitro. **Methods:** MCF-7 cells were exposed to leptin, leptin and compound and compound alone for 48 h. Cell viability was determined by 3-(4,5-dimethylthiazol-2-yl)-2,5-diphenyltetrazolium bromide MTT and fluorometric assays after 48 h incubation. Phosphorylation of ERK1/2 was quantified by ELISA. **Results:** Only quercetin, curcumin and EGCG showed significant protective effects against leptin-induced proliferation of MCF-7 cells. Increase in ERK1/2 phosphorylation in response to leptin was reduced by the addition of quercetin, curcumin and EGCG. **Conclusions:** Considering the high prevalence of obesity, this observation provides a rationale for use of curcumin, quercetin and EGCG as antagonists of leptin in the treatment of obese breast cancer patients.

## 1. Introduction

Breast cancer is the most commonly diagnosed cancer among women in the world but there are limitations in patient care mostly due to inability of predicting outcome and limited treatment options [1]. Several studies have indicated that levels of circulating metabolites such as adipokines, hormones, and cytokines are associated with breast cancer cell proliferation and migration [2,3]. Leptin is one such molecule considered to mediate breast cancer progression [4]. Leptin is a hormone mainly produced by white adipocytes. It is also synthesized by other organs/tissues including the mammary epithelium and placenta. Leptin plays an important role in energy homeostasis, food intake, reproductive processes, immunity, metabolism and oxidation of lipids, etc. [5]. Several studies have shown that leptin can exert proliferative effects on breast cancer cells in vitro [6,7,8,9]. Effects of leptin on mammary tumor cell growth included several mechanisms such as up/down regulation of apoptosis, anti-apoptotic genes and modulation of the extracellular environment [10]. Leptin receptor over expression has been observed in breast cancer compared to normal mammary epithelium [11,12]. Upon binding with its receptor, leptin activates pathways such as, Jak/STAT3, ERK1/2, phosphoinositide 3-kinase pathways and cyclin D1 expression [13]. In leptin-stimulated breast cancer cells, STAT3, extracellular signal-regulated kinase (ERK), and Akt activation has been demonstrated in several in vitro studies [14,15]. Several epidemiological studies have demonstrated that a diet rich in fruits and vegetables can reduce the risk of several cancers including breast cancer [16]. Many flavonoids, polyphenols, terpenoids, fatty acids, isothiocynates, proanthocyanidins and flavonolignans present in human diet have been shown to prevent cancer and are thus considered as possible chemopreventive agents [17,18]. Limited studies have been carried out to identify possible natural compounds which can inhibit the oncogenic role of leptin. Recently, protective effects of natural compounds, honokiol and benzyl isothiocyanate, in leptin-stimulated breast cancer cells have been demonstrated in vitro [8,19]. Indra et al. [20] have shown that quercetin, a natural polyphenol, can mediate protective effects in leptin-stimulated Human Umbilical Vein Endothelial Cells (HUVECs) in vitro.

Even though the therapeutic potential of inhibition of leptin action in disease conditions associated with metabolic syndromes is well recognized, inhibition of leptin signaling pathway/s in breast cancer has not been adequately investigated. Thus, the present study was designed to identify the protective effects of six dietary compounds (quercetin, curcumin, gallic acid, EGCG, ascorbic acid and catechin) against the oncogenic actions of leptin in MCF-7 breast cancer cells in vitro.

## 2. Materials and Methods

### 2.1. Cell Culture, Cell Culture Maintenance, Cell Culture Reagents and Chemicals

Human oestrogen receptor positive breast cancer cell line (MCF-7) and all the cell culture reagents were purchased from the American Type Culture Collection (ATCC, Manassas, VA, USA). MCF-7 cells were cultured according to the ATCC recommended culture conditions. Curcumin (C 1386), gallic acid (G 7384), epigallocatechin gallate (E 4143), ascorbic acid (A 4403) and catechin (43412) were purchased from Sigma-Aldrich, St. Louis, MO, USA. Quercetin and catechin were isolated from the bark of *Mangifera zeylanica* in one of our previous studies [21]. Human recombinant leptin was purchased from R&D systems (Minneapolis, Minnesota, USA, cat. no- 398-LP-05M).

### 2.2. Cell Viability Assays

#### 2.2.1. Determination of IC_50_ of Selected Compounds by MTT (3-(4,5-Dimethylthiazol-2-yl)-2,5-diphenyltetrazolium bromide) Assay

Upon 80% confluency, cells were harvested by trypsinization and seeded in 96-well plates at a density of 5000 cells per well. After a 24-h incubation period, cells were treated with the selected compounds (quercetin, curcumin, gallic acid, epigallocatechin gallate, ascorbic acid and catechin) and incubated for 48 h. MTT assay was then carried out as described by Gerlier and Thomasset [22].

#### 2.2.2. Effects of Letin on MCF-7 Cell Viability

MCF-7 cells were seeded in 96-well plates at a density of 20 × 10^3^ cells per well. After 12 h serum starvation, the culture media were changed to serum free media containing leptin (12.5, 25, 50, 100, 200 ng/mL) and incubated for 48 h. Doses of leptin were selected based on the studies carried out by previous researches [8,23]. Effects of leptin on cell proliferation were determined by MTT assay.

#### 2.2.3. Determination of Cell Viability in Leptin vs. Leptin and Compounds in MCF-7 Breast Cancer Cells by MTT Assay

To determine cell viability in leptin or leptin and compounds treated breast cancer cells, the method described by Indra et al. [20] was applied to MCF-7 breast cancer cells with modifications. Cells were seeded in 24-well plates at a density of 75 × 10^3^ cells per well. After 12 h serum starvation, the culture media were changed to serum free media containing treatments [(leptin (200 ng/mL); leptin (200 ng/mL) and compound (IC_50_ dose); compound alone (IC_50_ dose)]. The untreated controls only received serum free media. We assumed that the IC_50_ value obtained for six compounds from the MTT assay with 5 × 10^3^ cells would be marginally cytotoxic for 75 × 10^3^ cells. Therefore, the concentration of the compounds was maintained at IC_50_ dose (quercetin, curcumin, gallic acid, epigallocatechin gallate (EGCG), ascorbic acid and catechin). After 48 h incubation period, the effect of leptin or leptin with compounds on MCF-7 cell viability was determined by MTT assay. An amount of 200 ng/mL of leptin was chosen as this concentration showed the highest increase in MCF-7 cancer cell viability.

#### 2.2.4. Determination of Cell Viability by Fluorometric Assay

Cell viability reagent in the ApoTox-Glo™ Triplex Assay was used for further confirmation of results (effects of leptin on MCF-7 cell viability and determination of cell viability in leptin/leptin and compounds), according to the manufacturer’s instructions. This reagent measures cell viability using a fluorogenic, cell-permeate peptide substrate (glycyl-phenyl-alanyl-aminofluorocoumarin; GF-AFC).

### 2.3. Phospho-KinaseEnzyme-LinkedImmunosorbentAssay(ELISA)

Cell-based ELISA [Phospho-ERK1/ERK2 (T202/Y204) Immunoassay, R&D Systems, Minneapolis, Minnesota, USA (cat no. KCB1018),] was carried out according to the manufacturer’s instructions. Briefly, MCF-7 cells were grown and starved as mentioned in the previous section and treated with leptin, leptin and compound and compound alone for 48 h as indicated in methods 2. 2. 3 Two different fluorescent substrates were used for quantification. Fluorescence was measured on a Synergy™ HT Multi-Mode Microplate Reader; Bio-Tek Instruments, Inc., Winooski, VT, USA with excitation at 540 nm and emission at 600 nm (phosphorylated proteins) and excitation at 360 nm and emission at 450 nm (total proteins).

### 2.4. Statistical Analysis

At least three individual experiments in triplicate were carried out. Differences between the studied groups were determined by ANOVA, followed by Bonferroni test for selected pairs (GraphPad Software, Inc., La Jolla, CA, USA). All data are shown as means ± SD, and *p* <0.05 was considered as statistically significant.

## 3. Results

### 3.1. Cytotoxic Effects of Compounds in MCF-7 Cells

All the compounds used in the study showed cytotoxic properties in MCF-7 breast cancer cells after a 48-h incubation period. Among these, curcumin, ascorbic acid and epigallocatechin gallate showed the most potent cytotoxic effects after 48 h incubation whereas, catechin, quercetin and gallic acid showed less cytotoxicity in breast cancer cells. IC_50_ values of compounds are summarized in the Table 1.

### 3.2. Proliferation of MCF-7 Cells by Leptin Treatment

**Figure 1:**
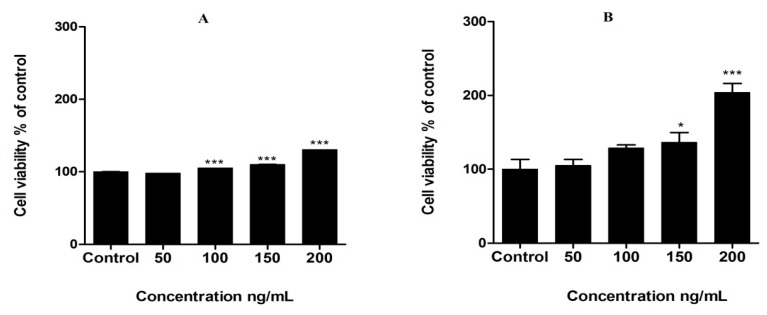
MCF-7 breast cancer cell viability after exposure to leptin for 48 h. (**A**) MTT assay (**B**) fluorometric assay. * *p* < 0.05, *** *p* < 0.0001 compared to the controls (one way ANOVA followed by Bonferroni test for selected pairs).

Leptin caused a significant dose-dependent increase in cell viability in MCF-7 breast cancer cells after 48 h incubation. Highest cell viability was observed at 200 ng/mL leptin concentration (*p* < 0.0001). Fluorometric assay also confirmed the effects of leptin in MCF-7 cell viability in a significant manner (*p* < 0.0001) (Figure 1).

### 3.3. Protective Effects of Tested Compounds against Leptin-Induced Breast Cancer Cell Proliferation

**Figure 2:**
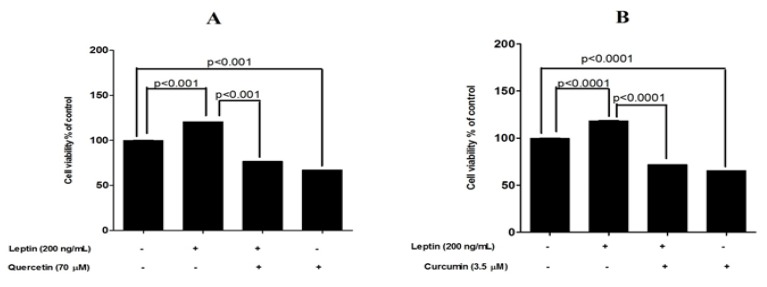

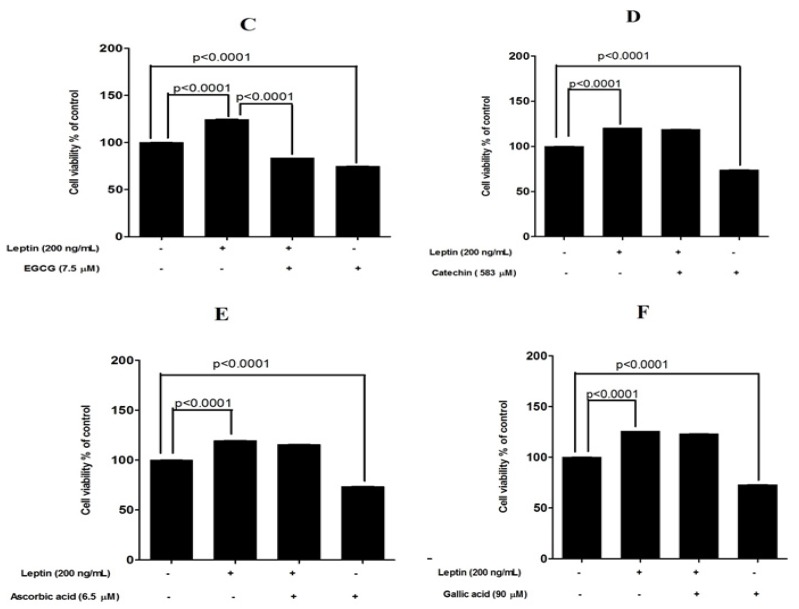
Protective effects exerted by six dietary compounds in leptin-stimulated MCF-7 breast cancer cells examined by MTT assay. (**A**) quercetin (**B**) curcumin (**C**) EGCG (**D**) catechin (**E**) ascorbic acid and (**F**) gallic acid. ** *p* < 0.001, *** *p* < 0.0001 compared with leptin-stimulated cells by one way ANOVA with Bonferroni test for selected pairs of columns.

**Figure 3:**
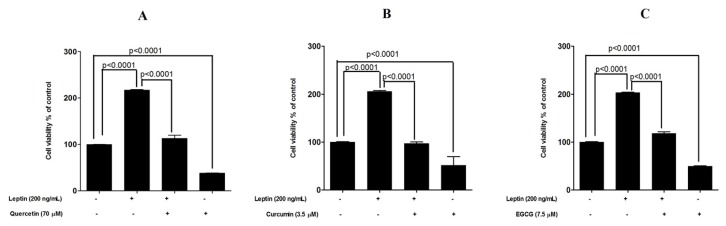
Protective effects exerted by (**A**) quercetin (**B**) curcumin and (**C**) EGCG in leptin-stimulated MCF-7 breast cancer cells examined by flurometric assay. *** *p* < 0.0001 compared with leptin-stimulated cells by one way ANOVA with Bonferroni test for selected pairs of columns.

In the experiment carried out with six selected test compounds, the addition of leptin increased the cell number by 18% to 25% at 48 h of exposure (MTT assay). Quercetin, curcumin and EGCG were able to reduce the basal cell proliferation by 25% to 35% (untreated cells vs. compound treated cells, *p* < 0.0001). In addition, quercetin, curcumin and EGCG were able to reduce the leptin-induced cell proliferation significantly (percentage reduction of 63% (*p* < 0.0001), 60% (*p* < 0.0001) and 66% (*p* < 0.0001) for quercetin, curcumin and EGCG respectively (Figure 2). Fluorometric assay also confirmed these results. However, this assay showed a higher cell proliferation in response to leptin with 103% to 117% increase in the cell number at 48 h incubation. Similarly, the reduction in basal cell proliferation in response to quercetin, curcumin and EGCG was 51% to 62% (untreated cells vs. compound treated cells, *p* < 0.0001). Quercetin, curcumin and EGCG were able to reduce the leptin-induced cell proliferation significantly (percentage reduction of 52% (*p* < 0.0001), 46% (*p* < 0.0001) and 58% (*p* < 0.0001) for quercetin, curcumin and EGCG respectively (Figure 3).

### 3.4. Inhibition of ERK1/2 Phosphorylation

**Figure 4:**
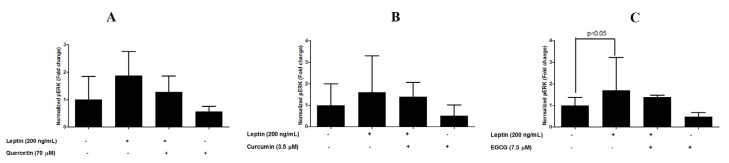
Leptin-induced ERK1/2 phosphorylation in (**A**) quercetin (**B**) curcumin and (**C**) EGCG in MCF-7 breast cancer cells.

Cell-based ELISA revealed that the addition of 200 ng/mL leptin increased ERK1/2 phosphorylation in MCF-7 cells by 60% to 87% at 48 h incubation. Quercetin, curcumin and EGCG were able to reduce basal ERK1/2 phosphorylation by 44% to 52% (untreated cells vs. compound treated cells). In addition, quercetin, curcumin and EGCG were able to reduce the ERK1/2 phosphorylation in leptin-stimulated cells (percentage reduction of 59%, 20% and 32% for quercetin, curcumin and EGCG respectively (Figure 4).

## 4. Discussion

Obesity is associated with many metabolic disorders and with an increased risk of developing breast cancer [2]. Though the link between obesity and breast cancer has not been exactly delineated, elevated leptin levels have been identified as one of the key factors for breast cancer development, progression and metastasis [2]. Evidence from several in vitro studies shows a proliferative effect of leptin on cancer cells including MCF-7 breast cancer cells [6,7,8,9]. In this study, we have also showed that leptin can increase the survival of MCF-7 breast cancer cells in a significant dose-dependent manner after 48 h incubation. Highest cell survival was observed at 200 ng/mL of leptin treatment in both the assays (colorimetric and fluorometric). However, the fluorometric assay showed a higher survival (2.03-fold increase) at 200 ng/mL leptin treatment than the MTT assay. This may be due to the greater sensitivity of fluorometric assays over colorimetric determinations [24].

A Number of phytochemicals with anti-obesity effects have been studied extensively. Among those, quercetin, curcumin, apigenin, luteolin, kaempferol, myricetin, quercetin, genistein, caffeine, daidzein, cyanidin, grape seed proanthocyanidin extract, xanthohumol, epigallocatechin gallate and resveratrol are some of the major compounds found to have anti-obesity effects [25]. Adipocytes cause increase of fat mass. Quercetin, epigallocatechin gallate, naringenin, rutin, hesperidin, resveratrol, naringin, genistein, p-coumaric acid, curcumin, ursolic acid, m-coumaric acid and chlorogenic acid have been identified as phytochemicals which specifically target adipocyte life cycle [25].

Jak/STAT3, ERK1/2, phosphoinositide 3-kinase pathways and cyclin D1 expression pathways are reported to be involved in mediating leptin-stimulated breast cancer cell growth [13]. Even though several compounds with anti-obesity properties have been identified, studies on natural compounds which can inhibit the oncogenic role of leptin and target leptin signaling pathway/s are limited. In the present study, it was observed that, quercetin, curcumin and EGCG can exert significant protective effects against leptin-induced MCF-7 breast cancer cells after 48 h incubation period. Though these three compounds are reported to possess anti-obesity properties, this is the first report on their protective role on leptin-induced breast cancer cells. Moreover; curcumin, quercetin and EGCG have also been identified as targeting the JAK-STAT pathway, which is one of the main leptin signaling pathways, in several cancer cells [26,27,28]. Several researchers have also demonstrated reduced leptin and leptin receptor gene expression in breast cancer cell lines after treatment with curcumin [29].

It has been reported that the ERK1/2 pathways play a major role in leptin signaling in MCF-7 breast cancer cells [30,31]. Effects of the compounds, found to be protective (quercetin, curcumin and EGCG) against leptin-induced breast cancer cells, on phosphorylation of ERK1/2 was assessed in the present study. Activation of ERK1/2 in response to leptin was confirmed by assessing the levels of phosphorylation of ERK1/2. Whether the compounds that exerted inhibitory effects on leptin-stimulated breast cancer cell proliferation reduce the ERK1/2 phosphorylation was also tested.Although the pERK1/2 levels were lower in cells co-treated with both the compounds and leptin than in the cells that received only leptin, the difference was not statistically significant. This is the first report on the identification of quercetin, curcumin and EGCG functioning as antagonists of leptin. However detailed studies on each compound are necessary to understand the underlying mechanism/s as antagonists of leptin.

## 5. Conclusions

This study provides experimental evidence of quercetin, curcumin and EGCG as potential dietary compounds which can inhibit leptin-induced MCF-7 breast cancer cell proliferation. Considering the high occurrence of obesity in the world, this study has the potential to influence obese breast cancer women by giving new opportunities for breast cancer research and eventually therapy using these three compounds against breast cancer.

## Figures and Tables

**Table 1:** IC_50_ values of six dietary compounds in MCF-7 cells examined by MTT (3-(4,5-Dimethylthiazol-2-yl)-2,5-diphenyltetrazolium bromide) assay after 48 h post incubation.

Compound Tested	IC_50_Values (µM)
Ascorbic acid	6.5
Catechin	583
Curcumin	3.5
EGCG	7.5
Gallic acid	90
Quercetin	70
